# The Role of Instagram in Orthopaedic Patient Education and Engagement: A Content Analysis From Singapore

**DOI:** 10.7759/cureus.83827

**Published:** 2025-05-10

**Authors:** Amirzeb Aurangzeb, Aloysius Ang Jian Feng, Dinesh Sirisena

**Affiliations:** 1 Orthopaedic Surgery, Changi General Hospital, Singapore, SGP; 2 Family Medicine, National University Health System, Singapore, SGP; 3 Sports Medicine, National University Health System, Singapore, SGP

**Keywords:** healthcare social media, orthopaedic sports medicine, orthopaedic surgery, patient education, social media engagement

## Abstract

Introduction

Instagram, the social media platform, has become a powerful medium for healthcare communication, particularly in patient education and engagement. This study examines how Instagram is utilised by orthopaedic surgeons in Singapore to disseminate health information and foster patient interaction.

Methods

A content analysis of publicly available Instagram posts from orthopaedic surgeons, clinics, hospitals, and professional bodies in Singapore was conducted. Posts published between March 1, 2023, and March 1, 2025, were categorised as educational, promotional, or engagement-focused. Key engagement metrics and thematic content were analysed.

Results

A total of 152 posts from orthopaedic accounts in Singapore were analysed. Educational posts constituted 76.3% of the content (n = 116), focusing on topics such as osteoporosis prevention and joint replacement surgery. Promotional posts accounted for 21.1% (n = 32), primarily advertising orthopaedic services, while patient engagement posts made up 2.6% (n = 4), including patient testimonials. Educational posts received the highest engagement (averaging 13.5 likes per post), followed by promotional (13.0 likes) and patient engagement posts (9.75 likes).

Conclusion

Instagram is a valuable platform for orthopaedic patient education and engagement in Singapore. The strong preference for visual educational content (e.g. infographics and procedural videos) aligns with Singapore’s multilingual society, where visual aids can transcend language barriers and improve comprehension. However, the prevalence of promotional content raises concerns about the commercialisation of healthcare on social media. This study underscores the need for local professional guidelines to optimise the use of Instagram in orthopaedic practice.

## Introduction

Social media has revolutionised the way healthcare professionals communicate with patients, offering unprecedented opportunities for patient education, engagement, and public health campaigns [1]. Among these platforms, Instagram has emerged as a particularly powerful tool due to its visual and interactive nature. This makes it ideal for disseminating health-related content, with its emphasis on visuals and interactivity. This is key for orthopaedic surgery, where imaging and procedural explanation are core to patient understanding [1,2]. Despite the advantages, the use of Instagram by healthcare professionals remains underutilised in some orthopaedic subspecialties [3,4]. However, concerns persist about misinformation, patient privacy and commercialisation, as seen in analyses of another popular social media tool, TikTok [5,6].

Singapore, with its high digital literacy and advanced healthcare system, provides a unique context for studying the role of Instagram in orthopaedic care. A total of 93.3% of physicians in Singapore owned a social media account, though it is unclear how many of them use social media for professional purposes. [7]. Hence, there is a paucity of local studies evaluating how orthopaedic professionals use these tools for patient education and engagement. This study addresses this gap by analysing Instagram posts from Singaporean orthopaedic professionals to evaluate their use for patient education and engagement.

## Materials and methods

Study design

This study employed a retrospective content analysis of publicly available Instagram posts from orthopaedic professionals and groups in Singapore. We noticed a few broad themes among the posts and hence divided them into three main themes: educational, promotional, and patient engagement (Table 1). Data was collected and analysed to understand how Instagram is used for orthopaedic patient education and engagement.

**Table 1:** Key Themes of Posts

Theme	Definition	Examples
Educational	Provides factual information about orthopaedic conditions, treatments, or prevention.	Infographics, videos, tips, and explanations of medical conditions/procedures
Promotional	Promotes orthopaedic services, products, or professionals.	Clinic advertisements, surgeon profiles, and event promotions
Patient Engagement	Encourages interaction or builds a sense of community.	Question & answer (Q&A) sessions, polls, quizzes, patient testimonials, and comments from followers

Data collection

Account and Posts Identification

Instagram accounts of orthopaedic surgeons, clinics, hospitals, and professional organisations in Singapore, as well as relevant posts, were identified using keywords related to Singapore and orthopaedics. Accounts were included if they met the following criteria: publicly available, based in Singapore or primarily serving a Singaporean audience, and posted content related to orthopaedics. Accounts were excluded if they were private or if their posts were unrelated to orthopaedics. Posts were included if they focused on orthopaedic conditions, procedures, patient education, clinic promotion, or patient engagement efforts. Posts published between March 1, 2023, and March 1, 2025, were reviewed. Only posts with visible engagement metrics were included so as to facilitate quantitative analysis. Posts unrelated to orthopaedics or outside the specified time frame were excluded. This study thus analysed all publicly available Instagram posts meeting the inclusion criteria during the defined observation period. The sample represents organic platform activity at the time of data collection.

Data extraction

Two independent reviewers performed the analysis for each post with the following data extracted: account name, post type (image, carousel, video, infographic), caption text and hashtags, engagement metrics (likes, comments, shares) and themes: educational, promotional, or patient engagement. Image posts were defined as static single-frame visuals such as clinical photographs or quote-style graphics with text overlays. Infographic posts were defined as designed visuals that consolidate or summarise information into a single image. Video posts were defined as posts that involved dynamic content with motion, whether live-action or animated. Carousel posts are multi-page posts that are navigated by swiping along a single post. This may take the form of combined images and infographics as well. In areas of uncertainty or contention with regard to the overall theme of the post, a third independent reviewer was engaged. Table 1 demonstrates the relevant themes used to categorise posts.

Data analysis

Both quantitative and qualitative analyses were performed in this study. Frequencies and percentages were calculated for post types and themes, with engagement metrics summarised using descriptive statistics. Qualitative thematic analysis categorised posts into the three predefined themes as per Table 1.

Ethics statement

No institutional review board or ethics approval was required for this study, as only publicly available data were analysed. No personal or private information was collected. Accounts and posts were anonymised in the analysis.

## Results

Post characteristics

A total of 152 Instagram posts from orthopaedic accounts in Singapore underwent thematic analysis and were classified into three different post themes (Table 2). The posts were further subdivided based on the different types: image, infographic, carousel or video (Table 3).

**Table 2:** Themes of Posts

Post Themes	Number of Post (n)	% of Total
Educational	116	76.30%
Promotional	32	21.10%
Patient Engagement	4	2.60%

**Table 3:** Types of Posts

Types of Post	Number of Post (n)	% of Total
Image	76	50%
Infographic	36	23.70%
Carousel	35	23.00%
Video	5	3.30%

Further breakdown and detailed analysis of the posts are shown in Tables 4-5. Educational posts dominated the dataset (76.3%, n = 116), focusing on topics such as osteoporosis prevention and joint replacement surgery. These posts received the highest number of mean likes per post (13.5). Promotional posts accounted for 21.1% (n = 32) and primarily highlighted orthopaedic services or surgeon expertise, garnering an average engagement of 13.0 likes per post. Patient engagement posts (2.6%, n = 4) included Q&A sessions and testimonials but had the lowest average engagement (9.8 likes per post). Visual aids, such as infographics (n = 36) and videos (n = 5), were frequently used in educational posts to explain complex concepts. Carousel posts (n = 35) were also popular, particularly for step-by-step procedural explanations or the use of multiple infographics within one post.

**Table 4:** Combined Post Type and Theme

Theme	Image	Video	Carousel	Infographic	Total
Educational	50	3	32	31	116
Promotional	22	2	3	5	32
Patient engagement	4	0	0	0	4

**Table 5:** Post Themes and Online Engagement Metrics

Theme	Total Number of Likes	Average Number of Likes	Total Number of Posts Shared	Total Posts with Comment
Educational	1565	13.5	2	1
Promotional	285	13.0	0	0
Patient engagement	39	9.8	0	0

As seen in Table 6, carousel posts had the highest average number of likes (29.2), followed by infographics (13.5), video (5.4) and then image (4.7). The average number of comments and shares was persistently low across all types of posts.

**Table 6:** Post Types and Online Engagement Metrics

Post Type	Total Number of Likes	Average Number of Likes	Total Number of Shares	Average Number of Shares	Total Number of Comments	Average Number of Comments
Carousel	1021	29.2	0	0	0	0
Image	356	4.7	0	0	0	0
Infographic	485	13.5	0	0	0	0
Video	27	5.4	5	1	2	0.4

Themes

While quantitative metrics such as likes, shares, and post frequencies provide valuable insights into engagement patterns, qualitative analysis is essential for interpreting the underlying context and meaning of the observed trends. In this study, qualitative methods were employed to categorise posts into three predefined themes: educational, promotional, and patient engagement. This allows for further analysis of the nuances of content that quantitative data alone could not capture. This approach aligns with established methodologies in social media research, as recommended by Jeyaraman et al., who recommended that data in social media research should undergo content analysis [1].

Educational Content

Educational posts covered a diverse range of orthopaedic topics, from prevention and management of sports injuries to arthritis management and spinal health. These posts frequently employed visual aids in the form of infographics, carousels or videos to enhance comprehension. Educational content garnered the highest engagement with the highest number of average likes per post.

Promotional Content

Promotional content served to highlight the expertise and achievements of orthopaedic surgeons, including awards and recognitions from professional bodies. This also included direct advertisement of clinical services such as advanced technologies or patient-centric amenities (short wait times, fast appointment booking, etc.). While these posts garnered high engagement (13.0 likes per post), their commercial tone might have risked overshadowing educational value.

Patient Engagement

Patient engagement included the form of testimonials from recovering/recovered patients sharing their personal experience. This often includes emphasis on pain relief or restored mobility, humanising care, and not addressing potential challenges such as rehabilitation setbacks.

## Discussion

Our analysis of 152 Instagram posts from various orthopaedic accounts in Singapore (March 2023 to March 2025) revealed three dominant themes: educational (76.3%), promotional (21.1%), and patient engagement (2.6%). The findings of this study offer important insights into the evolving role of Instagram in orthopaedic practice in Singapore's unique healthcare landscape. The predominance of educational content (76.3%) suggests that Singaporean orthopaedic professionals are primarily using Instagram as an educational platform rather than purely for marketing purposes. This aligns with the professional ethos of medicine while addressing the growing patient demand for accessible health information [1].

The superior performance of carousel and infographic formats (average number of likes 29.2 and 13.5 per post) has significant implications for health communication strategies. While the absolute number of likes per post was modest (<100), the relative engagement differences between content types were substantial and consistent, suggesting a preference for visually structured educational content among followers. These findings support Mayer’s Cognitive Theory of Multimedia Learning, which posits that visual representations enhance information processing and retention [8]. For orthopaedic conditions where spatial understanding is crucial (e.g. joint anatomy or surgical procedures), these visual formats may be particularly effective.

Interestingly, promotional posts made up 21.1% of the total dataset (n = 32) and garnered significant engagement with an average of 13.0 likes per post. This strong engagement suggests that promotional content, such as surgeon profiles, clinic services, or milestone achievements, may hold substantial appeal for followers. This aligns with findings from Gary and Samtani et al., who note that social media is increasingly used for personal branding and marketing in orthopaedic practice [2,9]. One possible explanation is that promotional posts tend to highlight success stories or showcase recognisable figures in orthopaedics, which may drive higher engagement through familiarity, aspirational content, or social proof. While these posts do not directly contribute to patient education, they may indirectly foster trust and interest in the healthcare provider, underlining the dual role of Instagram as both a medical and marketing tool. However, this raises important ethical considerations, as high engagement with promotional content may incentivise more advertising-driven posts at the expense of informative, patient-centred content [10].

Patient engagement posts, such as testimonials, were the least common (n = 4) and had the lowest average engagement (9.8 likes per post), compared to educational (13.5 likes) and promotional content (13.0 likes). This suggests that audiences may be less responsive to patient-centred narratives on Instagram, or that such content is less prioritised by content creators. The low frequency of these posts may reflect underlying concerns among healthcare providers about patient privacy, consent, and potential medico-legal risks. It may also indicate a preference for one-way information delivery over interactive or personal storytelling in this context.

The ethical and professional challenges associated with using Instagram in orthopaedic practice cannot be overlooked. There is a need to ensure quality control and regulation of healthcare information as per Jeyaraman et al. [1]. This concern is amplified by Gabarron et al., who systematically reviewed COVID-19 misinformation and found its prevalence ranged from 0.2% to 28.8% across platforms, with all included studies reporting that such misinformation generated public fear or panic [1,11]. This underscores a universal challenge for medical specialities, including orthopaedics, where unverified treatment claims could similarly influence patient decisions and outcomes. Moreover, the lack of professional guidelines with regard to social media practice increases the risk of breaching patient privacy and confidentiality, especially when testimonials or clinical images are shared. As highlighted by McLawhorn et al. and Jeyaraman et al., responsible digital conduct and informed consent are paramount to preserving public trust [1,12].

Our findings also highlight important differences from global trends. To our knowledge, this is the first study quantifying the use of Instagram in the context of Orthopaedic Surgery in Singapore. While prior work notes varied surgeon motivations for social media (e.g. education, marketing), regional comparisons remain limited by data gaps [2,4]. Our findings highlight Singapore’s strong emphasis on patient education, which may be due to Singapore’s unique medico-cultural context, where there is a nationwide shift in increasing healthcare literacy [13]. This thus emphasises the need for evidence-based practice with direct patient involvement in care, factoring in the existing multicultural patient population with diverse information needs. Singapore’s orthopaedic community appears to be leveraging Instagram effectively, likely due to the country’s high digital literacy and advanced healthcare infrastructure. However, more can be done to improve the poor patient engagement metrics observed in this study, such as incorporating interactive content (e.g. polls, Q&A sessions, interactive quizzes) or encouraging comments through prompts and open-ended questions. These strategies may potentially help foster two-way communication to enhance patient participation via increased organisation-audience interaction [14].

There is a need for continued research in this field, such as longitudinal studies or patient perspective studies on social media preferences. While this study provides valuable insights into Instagram's role in orthopaedic patient education and engagement, several limitations must be acknowledged. First, the analysis was restricted to publicly available posts, potentially excluding private accounts or direct messaging interactions that may represent additional forms of engagement. Second, the study focused solely on Instagram, omitting other platforms (e.g. Facebook/TikTok) that may also play significant roles in healthcare communication. Third, the content analysis was limited to quantitative metrics (e.g. likes, comments and shares) and thematic categorisation, without assessing the accuracy or quality of medical information presented in detail - a critical consideration given the risks of misinformation. Fourth, engagement metrics (such as the number of likes on a post) may not necessarily correlate with meaningful educational impact or behavioural change, as they could simply reflect passive viewing rather than genuine understanding or application of the information. Fifth, the study did not evaluate patient perspectives or behavioural outcomes, meaning the actual impact of these posts on health literacy or decision-making remains unclear. Finally, the study’s focus on Singapore may limit applicability to other regions with differing healthcare systems, cultural attitudes toward social media, or digital literacy levels. Future research should address these gaps by incorporating mixed-methods approaches, including patient surveys and cross-platform analyses, to provide a more comprehensive understanding of social media’s role in orthopaedic care. Such research may also explore the differences in social media engagement between public and private institutions.

This study serves as a critical first step in that direction, providing valuable insights into the current landscape and paving the way for future research and innovation. However, the lack of local studies on this topic underscores the need for further research to develop context-specific guidelines and best practices for integrating social media into orthopaedic care.

## Conclusions

This study highlights Instagram’s potential as a powerful tool for orthopaedic patient education and engagement in Singapore, with a predominance of educational posts (76.3%) and higher engagement with visual formats such as carousels and infographics. While absolute engagement levels were modest, the consistent trend across post types offers measurable insights into audience preferences. The low rate of patient engagement and the presence of promotional content raise important ethical and practical considerations. Our findings support Instagram’s potential as a supplementary tool for patient education but underscore the need for more robust data, particularly around patient impact and content accuracy. Local guidelines and further mixed-methods research are essential to ensure the responsible and effective integration of social media into orthopaedic care. As social media becomes integral to healthcare communication, orthopaedic professionals must balance informative and promotional content while safeguarding patient trust and privacy.
